# Enhanced Aging Stability of Ordered Mesoporous Silica Materials Synthesized via True Liquid Crystal Templating—A Small-Angle X-Ray Scattering Study

**DOI:** 10.3390/ma19101923

**Published:** 2026-05-08

**Authors:** Xiangyin Tan, Boshra Atwi, Huy Bui Duc, Michael R. Buchmeiser, Frank Giesselmann

**Affiliations:** 1 Institute of Physical Chemistry, University of Stuttgart, Pfaffenwaldring 55, 70569 Stuttgart, Germany; xiangyin.tan@ipc.uni-stuttgart.de (X.T.); huy.bui-duc@ipc.uni-stuttgart.de (H.B.D.); 2 Institute of Polymer Chemistry, University of Stuttgart, Pfaffenwaldring 55, 70569 Stuttgart, Germany; boshra.atwi@ipoc.uni-stuttgart.de (B.A.); michael.buchmeiser@ipoc.uni-stuttgart.de (M.R.B.)

**Keywords:** SBA-15, water adsorption, SAXS, nitrogen physisorption, structural aging

## Abstract

The long-term structural stability of ordered mesoporous silica (OMS) materials—specifically the durability of their pore architecture and pore lattice over time—is critical for their performance in catalysis, separation, drug carrier and nanoconfinement applications. SBA-15-type silica materials are synthesized via two different routes, namely the conventional “liquid crystal templating” (LCT) and the much less common “true liquid crystal templating” (TLCT) of micellar structures formed by the amphiphilic block copolymer Pluronic P123 in water. Here, we report that SBA-15 materials age very differently depending on the synthesis route: Under ambient conditions, OMS materials obtained via the LCT route undergo significant structural changes over time, especially in microporous regions, while SBA-15 materials obtained via the TLCT route show less or even no structural changes even after a year of storage. We attribute this enhanced aging stability of TLCT materials to their narrow mesopore size distribution and their much lower degree of microporosity.

## 1. Introduction

Ordered mesoporous materials (OMMs) are characterized by well-defined pore structures with diameters in the range of 2–50 nm, according to IUPAC classification [1]. These pores are arranged in long-range periodic frameworks. Common mesostructures include two-dimensional (2D) hexagonal arrays (space group P6/mmm), three-dimensional (3D) body-centered cubic (Im$\bar{3}$m), face-centered cubic (Fm$\bar{3}$m), and hexagonal close-packed (P6/mmc) symmetries [2,3]. The 2D hexagonal phase is the most common and wide-spread among all these different structures. OMMs are widely used in many important applications, such as heterogeneous catalysis, drug delivery, and molecular separation techniques [4,5].

**Figure 1:**
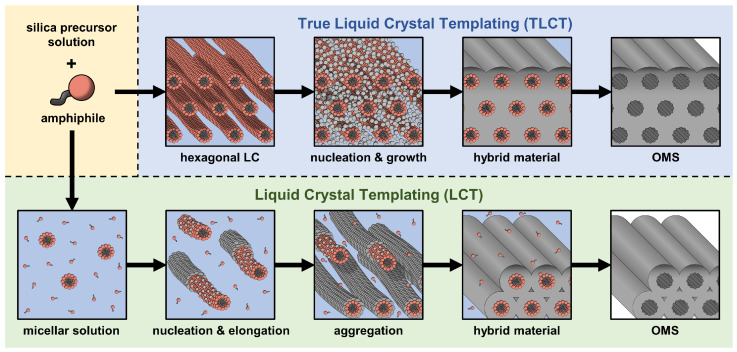
Synthesis routes to ordered mesoporous silica (OMS). Schematic illustration of the liquid crystal templating route (LCT, light green background) and the true liquid crystal templating route (TLCT, light blue background).

Important examples of OMMs are the ordered mesoporous silica (OMS) materials such as MCM-41 [6] and SBA-15 [7]. These OMS materials are mainly synthesized by using surfactants as structure directing agents via two different routes: The first route is called “true liquid crystal templating” (TLCT). In this method, the surfactant concentration is high enough to form a thermodynamically stable lyotropic liquid crystal phase, namely the hexagonal H_1_ phase. In this phase, rod-like surfactant micelles arrange themselves on a 2D hexagonal lattice, while water and the silica precursor fill the space between them. In these water-rich regions between the micelles, the silica precursor hydrolyzes into silicic acids which then progressively condensate to form a solid matrix of amorphous silica via the nucleation and growth of colloidal silica. In the hybrid material, these silica walls essentially fill the entire space between the micelles (Figure 1, upper row).

The second route is called “liquid crystal templating” (LCT) or “cooperative self-assembly” (Figure 1, lower row). Here, the surfactant concentration is so low that instead of a stable lyotropic liquid crystal phase, an isotropic solution of spherical surfactant micelles is formed. Under the influence of the silica precursor, which precipitates on the micelle surfaces, the micelles elongate from a spherical to a cylindrical shape and then gradually aggregate to form a 2D hexagonal structure (Figure 1, lower row). Once the aggregates are big enough, the hybrid material precipitates. However, even if the silica shells surrounding the micelles were perfectly cylindrical, their closest packing (during aggregation) does not result in silica walls that completely fill the space between the micelles in the hybrid material. It thus seems plausible that the silica walls formed via LCT exhibit more voids and micropores than in the case of TLCT. However, during the whole process, there is no stable liquid crystal formation [8] (as shown in Figure 1).

The aging stability of OMS materials is a critical concern for their long-term performance. Burleigh et al. studied the aging effect of OMS (using Brij-76 as the surfactant template) aged for 10 months under ambient environment without temperature and humidity control and found the lattice constant to decrease [9]. The lattice shrinkage occurring in calcined MCM-41 after storage in a shelf for 12 years was reported by Adeniran et al. [10]. Zhang et al. investigated the hydrothermal stability of SBA-15 in a steam environment at 600 °C for varying durations (from 3 to 24 h) and found that the unit cell parameter *a* decreased by 7.1%, i.e., from 11.3 nm to 10.5 nm after steaming [11]. However, capillary condensation occurring in SBA-15 and MCM-41 would not be accompanied by unit cell shrinkage; on the contrary, a slight increase in the pore lattice constant was observed [12,13]. Gor et al. reviewed recent advances in adsorption-induced deformation in nanoporous materials and discussed the mechanisms responsible for this phenomenon [14]. These findings suggest that the aging behavior observed in the present system may originate from adsorption-induced structural deformation.

In connection with a wider research project, the Collaborative Research Center (CRC) 1333 [15], in which several research groups from various disciplines are studying confinement effects in molecular heterogeneous catalysis, numerous OMS materials with a range of well-defined mesopore diameters were synthesized using both the LCT and TLCT routes. Typically, the freshly synthesized OMS materials are characterized after calcination using small-angle X-ray scattering (SAXS) and gas physisorption analysis and then stored until further use in a glass vial under ambient conditions in the lab. In this context, we noticed that OMS materials age very differently depending on the synthesis route: Under ambient conditions, OMS materials obtained via the LCT route undergo significant structural changes over time, as has also been reported previously. In contrast, OMS materials obtained via the TLCT route show virtually no structural changes even after a year of storage.

In this paper, we examine this remarkable observation in more detail, looking at the structural changes observed in LCT-based OMS materials after storage, and the reasons why these changes are not found in TLCT materials. The key for understanding this important difference between LCT- and TLCT-based OMS materials seems to be the presence of micropores, which are far less frequently found in TLCT-based OMS materials.

## 2. Materials and Methods

### 2.1. Material Synthesis

Pluronic P123 (average molar mass $\approx 5800\;g\;{\mathrm{mol}}^{-1}$), tetraethyl orthosilicate (TEOS, 98%), and tetramethyl orthosilicate (TMOS, 98%) were purchased from Sigma-Aldrich (Taufkirchen, Germany) and used as received without further purification.

We synthesized SBA-15 via liquid crystal templating (LCT SBA-15) according to the following procedure [16] by dissolving 16 g of Pluronic P123 in a mixture of 520 mL deionized water and 80 mL of 37 wt% hydrochloric acid. The solution was stirred overnight at 100 rpm in a 1 L Teflon-lined autoclave (Berghof Products + Instruments GmbH, Eningen unter Achalm, Germany) at room temperature. The temperature was then raised to 318 K, followed by the addition of 37 mL of tetraethyl orthosilicate (TEOS) as the silica source. The mixture was stirred at 318 K for 7.5 h at 150 rpm then subjected to hydrothermal treatment under static conditions at 353 K for 15 h. The precipitated product was recovered by vacuum filtration, washed thoroughly with deionized water, and dried in an universal oven (Memmert, Schwabach, Germany) at 353 K. To remove organic residues and open the pore structure, the as-synthesized SBA-15 was calcined (Muffle furnance L 9/11/SKM, Nabertherm, Lilienthal, Germany) at 823 K with an airflow of 150 L/h and a heating rate of 1 K/min.

We also synthesized the SBA-15 via true liquid crystal templating (TLCT SBA-15) as follows [8]: 8.98 g Pluronic P123 was weighed into a polypropylene tube and brought to the bottom of the container by heating it to approximately 323 K in a water bath. The surfactant-to-water ratio was chosen to be 50 wt%. In a separate polypropylene tube, 8.98 g of hydrochloric acid (0.1 M) were mixed with 12.65 g TMOS. The molar ratio of water to TMOS was 6. The mixture was stirred under reduced pressure (180 mbar) at 308 K for approximately 6 min to remove the methanol formed in the hydrolysis of TMOS. The mixture was then added to the P123 and stirred with a KPG stirrer (Eurostar 20, IKA, Staufen im Breisgau, Germany) for 10 min. After stirring, the liquid was transferred to PTFE dishes and placed in an oven at 353 K for two days. The formed hybrid material was ground and finally calcined to remove the surfactant by heating to 823 K for 6 h with a heating rate of 1 K/min and an air flow of 14.5 L/h. A summary of all samples is shown in Table 1.

### 2.2. Gas Physisorption Analysis

A Brunauer–Emmett–Teller (BET) analysis was performed using a Quantachrome Autosorb IQ device to determine the surface area, pore volume, and pore size distribution. Prior to measurement, the sample (30–50 mg) was placed in the cell and degassed for 12 h at a given temperature under vacuum. Afterwards, physisorption measurements were carried out using N_2_ at 77 K. For data collection and analysis, the QuadraWin Data Acquisition and Reduction Software, Version 7.1 was used. Two different evaluation models can be applied to estimate the pore size and pore size distribution, namely the Barrett–Joyner–Halenda (BJH) theory and density functional theory (DFT). BJH is incompatible for materials with small mesopores (<10 nm) such as MCM-41, for which DFT methods such as NLDFT (non-linear density functional theory) are more accurate [16]. Therefore, the surface area and pore size distribution were obtained from the adsorption branch via non-linear density functional theory (NLDFT) for the nitrogen sorption on silica for cylindrical pores. The pore radii ($r_{BET}$) of the different samples are shown in Table 2.

### 2.3. X-Ray Diffraction Analysis

Small-angle X-ray scattering (SAXS) measurements of mesoporous silica samples filled into Mark capillaries with a path length of 0.9 mm (Hilgenberg, Malsfeld, Germany) were performed using a Xeuss 3.0 SAXS instrument from Xenocs (Grenoble, France). The rectangular X-ray beam was generated by a Genix 3D source with a double slit collimation, operating with a copper anode, producing Cu $K_{\alpha }$ radiation with a wavelength of λ=1.5418 Å. The scattered intensity was recorded using a Dectris 2D EIGER2S 1M 2D-detector (Dectris AG, Baden-Daettwil, Switzerland). Measurements were performed with a beam size of $0.5\cdot 0.5\;{\mathrm{mm}}^{2}$, and at different sample-to-detector distances to allow a wide *q*-range and achieve improved resolution in *q*. Details can be found in the Supplementary Materials. Before measurement, the sample-to-detector distance was calibrated using a lanthanum hexaboride (LaB_6_) standard. For each detector position, the exposure time was 1 h. The final SAXS profiles were obtained by subtracting the background scattering from an empty capillary and subsequently merging the datasets from both detector distances into a single scattering curve. The effective thickness for absolute intensity was calculated from the Beer–Lambert law, $t_{\mathrm{eff}}=-\log T/\mu_{l}$, given the values of the linear absorption coefficient $\mu_{l}=77.37\;{\mathrm{cm}}^{-1}$ in the case of silica with mass density $2.2\;g\cdot {\mathrm{cm}}^{-3}$. The transmission *T* of the sample is calculated as the ratio of the fluxes transmitted through the capillary filled with the sample and through the empty capillary alone. Data reduction and processing were performed using the XSACT software (v2.10) package provided by Xenocs. The SAXS curves were analyzed using the well-established SBA-15 scattering model proposed by Sundblom et al. [17,18], and some relevant values of the output fitting parameters are shown in Table 2. The complete sets of fit parameters from the SBA-15 model are summarized in the Supplementary Materials.

**Figure 2:**
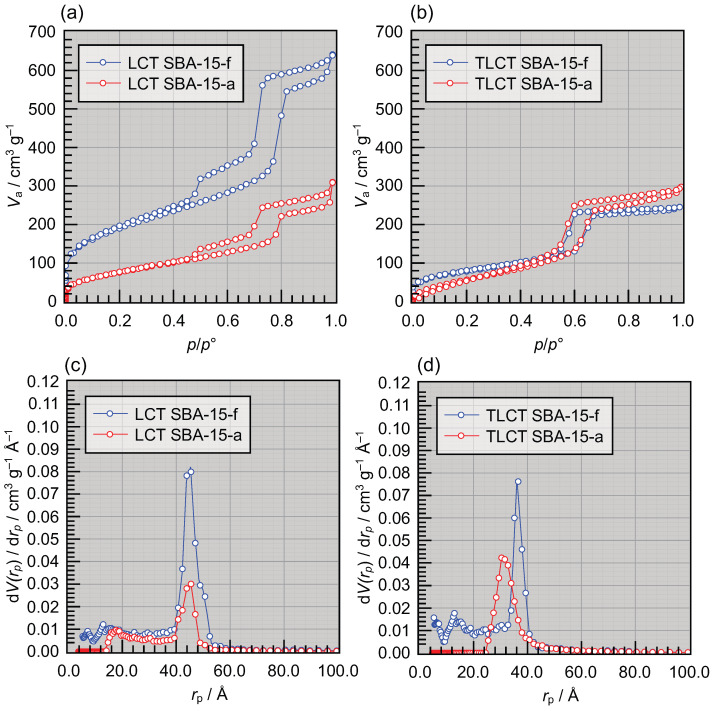
Nitrogen physisorption isotherms (specific adsorbed N_2_-volume $V_{a}$ vs. relative N_2_ pressure $p/p^{\circ }$) of (**a**) fresh and aged SBA-15 synthesized via liquid crystal templating (LCT) and (**b**) fresh SBA-15 synthesized via true liquid crystal templating (TLCT). (**c**) Incremental pore size distributions $dV\left(r_{p}\right)/dr_{p}$ vs. the pore radius $r_{p}$ derived from the adsorption branches in (**a**). (**d**) Incremental pore size distribution derived from the adsorption branch in (**b**).

**Figure 3:**
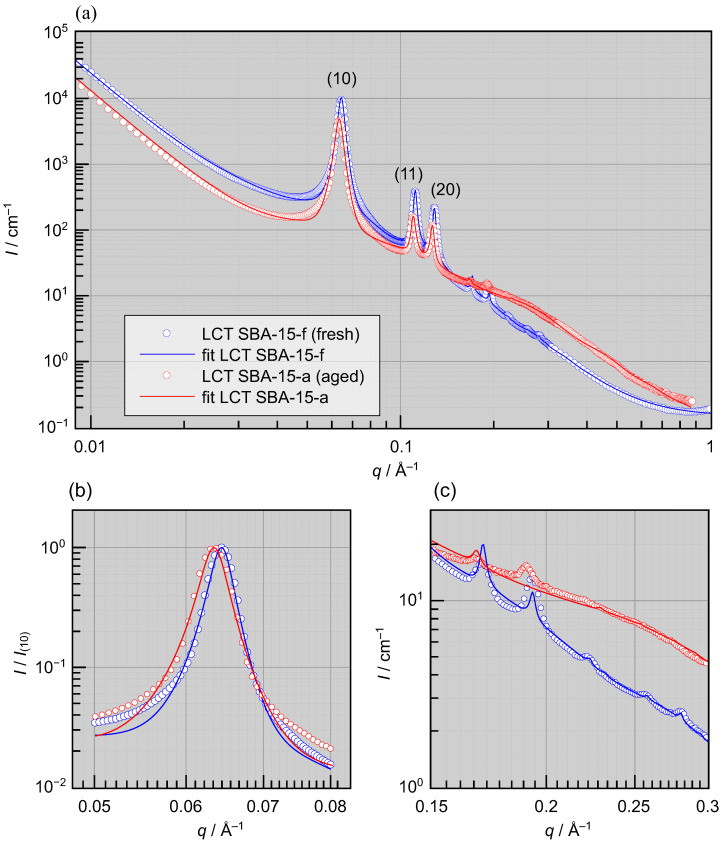
Small-angle X-ray scattering (SAXS) profiles illustrating the aging behavior of mesoporous SBA-15 synthesized via liquid crystal templating (LCT). (**a**) The absolute scattered intensity I(q) is plotted vs. the magnitude of the scattering vector *q*. Data sets for the freshly calcined sample SBA-15-f and for the aged sample SBA-15-a are shown together with the corresponding fit curves according to the SBA-15 scattering model in [17,18]. (**b**) The detailed view highlights the shift of the (10) Bragg peak as a result of aging; the intensities I(q) are normalized to the respective peak maxima $I_{\left(10\right)}$ to facilitate comparison of peak positions and shapes. (**c**) The figure magnifies the high-*q* region dominated by micropore scattering, emphasizing the aging-induced changes in this *q*-range. Sample abbreviations are defined in Table 1.

**Figure 4:**
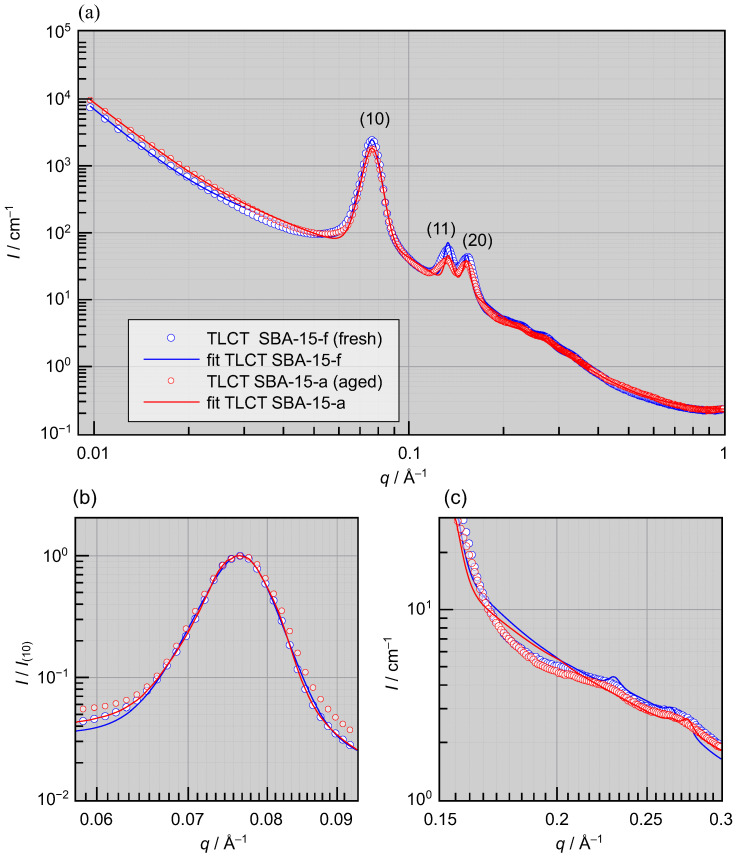
Small-angle X-ray scattering (SAXS) profiles (**a**) of absolute scattered intensity *I* vs. scattering vector *q* of SBA-15 synthesized via true liquid crystal templating (TLCT). Even after one year of aging under ambient conditions, no significant changes are observed in either the leading (10) Bragg peaks (**b**) or in the micropore scattering (**c**). Sample abbreviations are defined in Table 1.

## 3. Results

In this section, we first provide an overview of the experimental observations and then a detailed analysis and discussion of these results.

### 3.1. Experimental Observations

Nitrogen physisorption isotherms of the LCT- and TLCT-derived SBA-15 materials are shown in Figure 2 together with the corresponding pore size distributions. In Figure 2a,b, all measured materials show a type IV(a) isotherm which is typical of mesoporous materials [19]. LCT- and TLCT-derived materials, however, differ in the characteristics of their adsorption hysteresis. While the TLCT material shows a regular H1 loop, which indicates a narrow range of uniform mesopores, the LCT material shows a rather unusual H5 loop corresponding to both open and partially blocked mesopores [19]. In Figure 2a we observe a significant change in both the size and height of the loops after storing the LCT-derived OMS in ambient environment. In contrast, under the same storage conditions, there are almost no changes observed in the adsorption hysteresis loops of TLCT SBA-15 (see Figure 2b). BET theory was applied to calculate the specific surface area in the typical $p/p_{0}$ range between 0.05 and 0.3 [20]. The obtained data are listed in Table 2.

To examine the structural changes associated with the aging of LCT- and TLCT-derived SBA-15 materials, SAXS measurements were performed. The resulting SAXS profiles are presented in Figure 3 and Figure 4.

As already seen in the adsorption isotherms, significant structural changes are observed in the case of LCT-derived SBA-15 in Figure 3. The freshly calcined LCT SBA-15-f exhibits up to eight well-defined Bragg peaks, indicative of a highly ordered two-dimensional hexagonal arrangement of the mesopores. However, after one year of aging, significant changes can be seen in the SAXS profiles shown in Figure 3:

- On the one hand, at higher values of *q* corresponding to the length scales of micropores, a strong increase in scattering intensity is observed. This observation suggests that, the microporosity increased during aging. As a result of this increase in micropore scattering, the weakest Bragg reflections are no longer seen in the aged sample.
- On the other hand, the Bragg peaks have slightly shifted to smaller values of the scattering vector *q*, which is particularly evident in the leading (10) peak seen in Figure 3b (as well as in the higher order peaks). This shift indicates an expansion of the 2D hexagonal lattice during aging.

In clear contrast, the TLCT-derived SBA-15 does not show any significant change in the SAXS profiles (Figure 4a) in either the position and shape of the (10) Bragg peak (Figure 4b) or in the micropore scattering (Figure 4c).

All in all, we can conclude even from these qualitative considerations that TLCT-derived SBA-15 is obviously more resilient to aging than LCT-derived SBA-15. The reasons behind this remarkable difference will be discussed below, and it seems that the presence of micropores is the key to its understanding. Relevant parameters of the materials under investigation are summarized in Table 2.

### 3.2. Detailed Analysis and Discussion

We start with the NLDFT analysis of nitrogen physisorption. The BET surface area obtained for the freshly calcined LCT SBA-15-f was 620 m^2^/g, and it was reduced by almost a factor of three to 230 m^2^/g in the aged LCT SBA-15-a. LCT SBA-15-f shows a narrow pore size distribution in Figure 2c. After aging, a decrease in the mean peak maximum referring to a reduction in the pore volume was observed in LCT SBA-15-a, suggesting a partial collapse of the mesopores. Almost no change in the pore radius (ca. 45.6 Å) was observed.

In Figure 2a, a distinct step in the desorption isotherm is observed at $p/p^{\circ }\approx 0.45$ in the desorption branch caused by cavitation. A similar observation was already reported [21,22]. During the desorption step, the fluid evaporation starts in the large pores, while the evaporation is inhibited in the narrow neck or partially blocked pores. Once the relative pressure falls below the cavitation threshold, the adsorbed liquid is suddenly released from these pores, and, as a result, a sudden drop in $V_{a}$ is observed in the desorption isotherm in Figure 2a. Interestingly, the magnitude of this drop decreases throughout the aging process, suggesting that the narrow neck pores are partially closing.

LCT SBA-15-f shows a hysteresis that closes at a pressure range between 0.2 and 0.3. However, the hysteresis closure is shifted to $p/p_{0}$ around 0.4 in LCT SBA-15-a. The amount of adsorbed gas molecules at saturated pressure is higher in fresh samples, which indicates that the fresh materials exhibit higher surface area than the aged ones.

The SAXS curves are analyzed in terms of the scattering model of Sundblom et al. [17] which has been widely applied to SBA-15-type materials [18,23]. In the fitting, the (21) and (30) reflections are not reproduced as accurately as the lower-order peaks. The fitted intensity is higher in the case of the (21) peak but lower in the case of the (30) peak than the experimentally observed intensities (see Figure 3c). A similar mismatch has also been reported by Losito et al. [4], and it might originate from an imperfect combination of structure and form factors (see Supplementary Materials, Figures S5 and S6). In the SAXS fitting model, the total scattering intensity is separated into three main contributions, namely, the scattering from the macro-, meso-, and micropores. Porod’s law is used for the macropore scattering, and its exponent d≈4 remains essentially unchanged for all LCT samples (see Table 2), indicating a flat outer surface with negligible roughness. The micropore scaling factor $Sc_{micro}$, however, increases from 0.16 in the fresh LCT SBA-15-f to 0.36 in the aged LCT SBA-15-a, indicating a substantial increase of microporosity during aging.

According to our SAXS analysis, the pore radius of LCT SBA-15-f is 42.8 Å, which agrees well with the value obtained from physisorption. The same SBA-15 model, assuming empty mesopores, was applied to the aged sample (LCT SBA-15-a), as it remained essentially dry. However, the fitted pore radius is reduced by 3.3 Åcompared to the freshly calcined sample, suggesting that some of the mesopores may have adsorbed a small amount of ambient moisture.

Interestingly, the aged LCT-SBA-15 sample still retains a two-dimensional hexagonal structure, as the five leading diffraction peaks can still be indexed very well assuming p6/mm symmetry (see Supplementary Materials Figure S7). However, noticeable peak broadening is observed in LCT SBA-15-a, with the δ increasing by 24%, possibly reflecting a reduced domain size in the 2D lattice.

The SAXS peak positions used for the strain analysis are determined from the hexagonally ordered reflections of the mesoporous lattice. For a two-dimensional hexagonal lattice, the expected position of a reflection indexed by (hk) is

(1) $$q_{hk}^{m}=\frac{4\pi }{\sqrt{3}\;a}\sqrt{h^{2}+hk+k^{2}},$$

where *a* is the fitted hexagonal lattice parameter from the SAXS model. The fitted value of *a* for each sample is shown in Table 2. The uncertainty of the peak position $q_{hk}$ mainly originates from the uncertainty of the sample to detector distance δL, and, considering the scattering geometry, X-rays get scattered from the front and the back of the sample capillary. Thus, δL is given by the diameter of the capillary (0.9 mm), and $\delta q_{hk}=q_{hk}\frac{\delta L}{L}$. Detailed analysis of the uncertainties in q and ϵ is shown in the Supplementary Materials. For each reflection, a local peak window is defined around the fitted reference position. The initial half-width of the window is set by the fitted peak-width parameter δ:

(2) $$\Delta q_{\mathrm{win}}=3\delta .$$

Thus, for well-separated peaks, the window boundaries were

(3) $$q_{\mathrm{low}}=q_{hk}^{m}-3\delta ,\;q_{\mathrm{high}}=q_{hk}^{m}+3\delta .$$

For closely spaced higher-order reflections, this window can overlap with neighboring hexagonal reflections. To avoid assigning the same intensity region to adjacent peaks, 0.45 is used to prevent neighboring peak windows from overlap, and the half-width is capped by the nearest-neighbor reflection spacing:

(4) $$\Delta q_{\mathrm{win}}=\min\left[3\delta ,\;0.45\min\left(q_{hk}^{m}-q_{\mathrm{prev}}^{m},q_{\mathrm{next}}^{m}-q_{hk}^{m}\right)\right].$$

The final peak window is therefore

(5) $$q_{\mathrm{low}}=q_{hk}^{m}-\Delta q_{\mathrm{win}},\;q_{\mathrm{high}}=q_{hk}^{m}+\Delta q_{\mathrm{win}}.$$

Within this window, the peak positions are calculated via intensity weighting, denoted as $q_{\mathrm{cal}}$, calculated as the centroid of the measured intensity distribution inside the same window:

(6) $$q_{\mathrm{cal}}=\frac{\sum_{i}q_{i}I_{i}}{\sum_{i}I_{i}},\;q_{i}\in \left[q_{\mathrm{low}},q_{\mathrm{high}}\right].$$

The peak of the Miller index higher than (30) is very weak for most samples, so in the strain analysis, peaks higher than (30) are not used. This centroid-based value is used for the strain analysis because it uses the full peak shape within the defined window rather than relying only on a single maximum-intensity data point, and it can also try to avoid the influence of the SAXS model. The pore lattice strain for each sample is obtained from the average shift of all the $q_{\mathrm{cal}}$ as

(7) $$\varepsilon =\frac{\sum_{hk}\frac{q_{hk}^{ref}-q_{hk}^{com}}{q_{hk}^{ref}}}{n},$$

The resulting strain values are shown in the Table 3.

The strain of the LCT SBA-15 between fresh and aged ε is 1.53±0.05%, which is significantly larger than the values of about 0.3% reported previously for similar systems [24,25]. In contrast, the TLCT SBA-15 practically does not strain during aging, i.e., ϵ≈0, only a slight decrease in the intensity but no shift in $q_{cal}$ of the peaks are observed after one-year storage under ambient conditions, as shown in Figure 4. We attribute this enhanced structural robustness to the TLCT process, which should result in a more homogeneous and condensed silica matrix with less microporosity than the LCT mechanism, where the aggregation of irregularly shaped silica shells around the micelles could lead to defects and voids in the silica matrix (see Figure 1).

We now investigate the possible mechanism behind Bragg’s peak shift observed in the case of LCT-derived materials. Following the early suggestion of Zickler et al. [13], partially filled pores contract under capillary pressure and exert negative tensile forces on neighboring lattice sites, thereby increasing the inter-mesopore distance. Ludescher et al. and Gor et al. [14,25] discussed more recently that the peak shift might mainly originate from contrast-induced variations of the form factor of the pore. The maximum ϵ they reported in SAXS is about 0.3%. To examine this option, we performed SAXS simulations with different contrast ratios in the pores (see Figure S3), which lead in our case to maximum strains of 0.3% only. Furthermore, we removed the absorbed moisture by gently drying the LCT SBA-15-a under vacuum at 50 °C for a day. As a result, only a minor peak shift correction is observed (see Supplementary Materials Figure S1), and the average ϵ is −0.45±0.09%. Both calculations and experiments show that contrast effects alone would cause a strain of less than 0.4%, far smaller than the 1.5% observed. Furthermore, if the peak shift is caused solely by contrast variations, the higher-order Bragg reflections would remain at their original positions and would not shift coherently with the (10) peak (see Figure 3c). We thus conclude that the pore lattice expansion is the dominant reason for the *q*-shift of the Bragg peaks.

The quantitative relationship between the relative pressure $p/p_{0}$ (often referred to as relative humidity in the case of water vapor) and the radius of a liquid–vapor meniscus that can be stabilized within a cylindrical pore is expressed by the Kelvin equation [19]:

(8) $$\ln\left(\frac{p}{p_{0}}\right)=-\frac{2\gamma V_{m}}{RTr_{k}},$$

with the surface tension γ and the molar volume $V_{m}$ of the bulk fluid, the gas constant *R*, the temperature *T*, and the Kelvin radius $r_{K}$. Under typical environmental conditions in laboratory (18–23 °C and relative humidity of approximately 40–80%), water preferentially condenses in pores with radii below about 12–47 Å, and the full table of the condensate radii is shown in the Supplementary Materials, Table S4 [26]. This implies that, within the natural pore-size distribution of LCT-SBA-15-f (Figure 2b), the smaller mesopores can host capillary-condensed water, whereas the larger mesopores remain in a regime where only adsorbed multi-layers of water form on the inner-pore silica surface. The scattering intensity of LCT-SBA-15-a is higher than that of the fresh sample at $q>0.15\;{Å}^{-1}$, corresponding to real-space length scales of approximately d≈2π/q≈42Å.

This non-uniform water filling in the pores gives rise to a strongly heterogeneous capillary stress field inside the material. Capillary-condensed water in the small mesopores exert negative Laplace pressure on the silica walls. In contrast, the larger mesopores experience less or essentially no capillary pressure, which leads to expanding the neighbor pore lattice (see Supplementary Materials Figure S4) [14]. The silica framework is therefore subjected to an intrinsically unbalanced mechanical environment characterized by the concave meniscus water filled in samll mesopores adjacent to relatively stress-free large mesopores. Under such conditions, the silica network undergoes a four-step cycle that drives structural evolution. First, stress-assisted hydrolysis happens in the small mesopores where mechanical stress is highest, and Si–O–Si linkages are strained, making them more reactive. Water breaks these bonds to form silanol groups (Si–OH). Second, dissolution happens to the silanol group, and mobile silica species ($Si{\left(OH\right)}_{4}$) are formed [27,28]. Third, the capillary pressure drives $Si{\left(OH\right)}_{4}$ to big mesopores via the interconnected micropores within the wall [29]. During transport, silica precipitates in the micropores, new Si–O–Si bonds form, and finally some micropores might be closed. As an overall result of the mechanochemical rearrangements, the wall thickness *T* increases after aging which also implies an increasing center-to-center pore distance *a* (see Table 2).

Quantitative fitting of the micropore contribution used in the SAXS model reveals that the correlation length ξ of the Debye–Anderson–Brumberger (DAB) component decreases upon aging, indicating a fragmentation of extended micropore domains into smaller correlation units. At the same time, the micropore scaling factor $Sc_{\mathrm{micro}}$, which is proportional to the scattered intensity and thus to the volume fraction and contrast of microporous regions, increases. Interestingly, N_2_ physisorption does not show any increase in accessible micropore volume (Figure 2b). Instead, the micropore volume even slightly decreases. The apparent discrepancy between the SAXS results, which indicate an increase in microporosity, and the $N_{2}$ physisorption measurements, which indicate a decrease in micropore volume, can only be resolved by assuming that the newly formed micropores are closed or non-percolating, making them inaccessible to $N_{2}$ physisorption at 77 K. Such sealed voids contribute strongly to the SAXS contrast, as SAXS is sensitive to all electron density inhomogeneities irrespective of accessibility to the gas moleucule, but they do not contribute to gas adsorption. We find an interesting situation here in which neither SAXS nor gas physisorption alone provides an accurate picture. Rather, it is the combination of both methods that provides the (indirect) conclusion that aging induces a restructuring of the original micropore network into isolated or closed nanovoids.

The evolution from open micropores to closed nanovoids might probably be related to the mechano-chemical mass redistribution proposed earlier. The dissolution–reprecipitation cycle thickens the pore walls, seals parts of microporous channels, and generates closed voids within the corona. These processes are irreversible under the mild conditions studied here, thereby explaining both the loss of long-range order and the increase in the lattice parameter.

Our SAXS analysis reveals that TLCT templating results in a narrow mesopore size distribution (Table 2). This observation is consistent with earlier reports, which also demonstrate reduced pore-size dispersion and low microporosity in TLCT-derived SBA-15 materials [8,30]. Both features prevent the formation of strong stress imbalances since the pores condense water at nearly the same relative humidity. The small reduction in (10) peak intensity that was nevertheless observed may be related to the adsorption of atmospheric moisture, which reduces the scattering contrast at the inner pore wall. Upon drying (TLCT SBA-15-a), the (10) peak intensity increases slightly again (see Supplementary Materials Figure S3), indicating partial removal of the water; however, the peak does not fully return to its original state, suggesting that some small but irreversible changes remain in the mesopores even in TLCT derived materials.

## 4. Conclusions

The experimental results of this study show that SBA-15 materials, which were freshly produced and calcined using the standard LCT process, undergo irreversible aging when stored under ambient conditions for several months. The primary reason for this aging is most likely the presence of open micropores and comparatively broad pore size distribution. Capillary condensation of ambient humidity happens sequentially according to the pores’ diameter and leads to unbalanced mechanical stresses, which causes mechano-chemical rearrangements, increased closed voids, and distortions of the mesopore lattice in the long term. If, on the other hand, the SBA-15 materials are produced using the TLCT process, they exhibit much higher structural robustness against aging. We attribute this remarkable stability to the templating of an already ordered hexagonal liquid crystal phase, which is likely to result in a much more homogeneous silica matrix with a narrow mesopore size distribution and a significantly lower degree of microporosity. In the case of TLCT materials, capillary condensation thus creates much less heterogeneous stress in the material and thereby enhances its aging stability.

## Figures and Tables

**Table 1:** Sample information and abbreviations used in this study.

Abbreviation	Description
LCT SBA-15-f	SBA-15 synthesized via LCT, freshly calcined, and stored in a sealed 0.9 mm capillary
LCT SBA-15-a	SBA-15-f, stored in a glass vial with a snap-on plastic cap at ambient conditions for one year
LCT SBA-15-ad	SBA-15-a is gently dried under vacuum at 323 K for 24 h
TLCT SBA-15-f	SBA-15 synthesized via TLCT, freshly calcined, and stored in a sealed 0.9 mm capillary
TLCT SBA-15-a	TLCT SBA-15-f stored under the same conditions as LCT SBA-15-a
TLCT SBA-15-ad	TLCT SBA-15-a, gently dried under vacuum at 323 K for 24 h

**Table 2:** Some structural parameters obtained from the analysis of small-angle X-ray scattering (SAXS) profiles (Figure 2, Figure 3 and Figure 4) and corresponding gas physisorption (BET) measurements. Units: *a*, *r*, $\sigma_{r}$, *T*, ξ, and $r_{\mathrm{BET}}$ in Å; δ in ${Å}^{-1}$; $A_{s}$ in m^2^ $g^{-1}$. Parameters: *a*—lattice parameter; *r*—mesopore radius; $\sigma_{r}$—standard deviation of mesopore radius distribution; *T*—geometrical wall thickness (T=a−2r); δ—full width at half maximum of the diffraction peak; ξ—micropore correlation length; ${\mathrm{Sc}}_{micro}$—micropore scaling factor; $r_{\mathrm{BET}}$—pore radius from BET; $A_{s}$—specific surface area; *d*—Porod exponent. Uncertainties in the last digit are given in parentheses.

Sample	*a*	*r*	$\sigma_{r}$	*T*	δ	ξ	${\mathrm{Sc}}_{\mathrm{micro}}$	$r_{\mathrm{BET}}$	$A_{s}$	*d*
LCT SBA-15-f	112.58 (2)	42.8 (3)	2.4 (2)	27.0 (6)	$2.52\times 10^{-3}$ (2)	13.3 (2)	0.16 (4)	45.5	618.0	4.00 (6)
LCT SBA-15-a	114.33 (3)	38.7 (5)	4.7 (2)	36.9 (10)	$3.10\times 10^{-3}$ (4)	5.2 (2)	0.34 (1)	45.6	233.0	4.00 (1)
LCT SBA-15-ad	113.75 (5)	39.7 (7)	4.6 (2)	34.4 (14)	$3.56\times 10^{-3}$ (5)	5.7 (2)	0.28 (1)	–	–	4.00 (2)
TLCT SBA-15-f	94.45 (3)	33.9 (2)	1.9 (2)	26.7 (4)	$5.79\times 10^{-3}$ (3)	9.3 (1)	0.12 (1)	31.6	239.0	3.93 (4)
TLCT SBA-15-a	94.64 (3)	31.0 (3)	2.8 (2)	32.6 (6)	$6.34\times 10^{-3}$ (3)	8.3 (2)	0.12 (2)	30.4	241.4	3.63 (2)
TLCT SBA-15-ad	94.84 (3)	33.1 (2)	2.0 (2)	28.6 (4)	$5.53\times 10^{-3}$ (3)	9.1 (1)	0.12 (1)	–	–	3.86 (1)

**Table 3:** Strain calculated from intensity-weighted SAXS peak positions. Negative values indicate a shrinkage of the pore lattice. In TLCT SBA-15 the (21) peak is extinguished by a minimum in the form factor.

Comparison	Peaks Used	Average Strain (%)	Average Strain Uncertainty (%)
TLCT SBA-15 fresh vs. aged	(10), (11), (20), (30)	0.1	0.1
LCT SBA-15 fresh vs. aged	(10), (11), (20), (21), (30)	1.53	0.05
LCT SBA-15 fresh vs. aged degas	(10), (11), (20), (21), (30)	1.07	0.08
LCT SBA-15 aged vs. aged degas	(10), (11), (20), (21), (30)	−0.45	0.09

## Data Availability

The original data presented in the study are openly available in DaRUS, the University of Stuttgart data repository, at https://doi.org/10.18419/DARUS-5767.

## Supplementary Materials

The following supporting information can be downloaded at: https://www.mdpi.com/article/10.3390/ma19101923/s1, Figure S1: SAXS patterns of LCT SBA-15-f of the first peak, and the contrast changes inside the mesopore; Figure S2: SAXS patterns illustrating the aging behaviour of mesoporous SBA-15 synthesized via TLCT; Figure S3: SAXS patterns illustrating the aging behaviour of mesoporous SBA-15 synthesized via LCT; Figure S4: Schematic description of the lattice swelling; Figure S5: SAXS model fitting; Figure S6: Zoom of the Figure S5 where q>0.08Å; Figure S7: The Proof of 2D hexagonal symmetry in LCT SBA-15-a. Table S1: Summary of all parameters contained in the multi-scale model; Table S2: Sample names and corresponding sample-to-detector distances used in SAXS measurements; Table S3: Summary of multi-scale SAXS model parameters for various mesoporous silica samples; Table S4: Kelvin radius for capillary condensation calculated from the modified Kelvin equation for cylindrical pores; Table S5: Sample-to-detector distances used for the propagation of the geometric *q* uncertainty. References [17,31,32,33] are cited in Supplementary Materials.

## Abbreviations

The following abbreviations are used in this manuscript:

OMMs	Ordered mesoporous materials
OMS	Ordered mesoporous silica
LCT	Liquid crystal templating
TLCT	True liquid crystal templating
SAXS	Small angle X-ray scattering
BET	Brunauer-Emmett-Teller
DFT	Density functional theory
NLDFT	Nonlinear density functional theory
