# Supplementary material for: Enhanced Aging Stability of Ordered Mesoporous Silica Materials Synthesized via True Liquid Crystal Templating—A Small-Angle X-Ray Scattering Study
Source: Materials (Basel). 2026 May 8;19(10):1923. doi: 10.3390/ma19101923 (PMC13208761; doi:10.3390/ma19101923)
Supplement: Supplementary file 1 [file materials-19-01923-s001.zip › materials-4250549-supplementary.pdf]

Supplementary Materials: Enhanced Aging Stability of Ordered Mesoporous Silica Materials Synthesized via True Liquid Crystal Templating - a Small-Angle X-Ray Scattering Study

Xiangyin Tan [1], Boshra Atwi [2], Huy Bui Duc [1], Michael R. Buchmeiser [2], Frank Giesselmann [1*]

S1. SBA-15 Model Equations and Parameters

The SAXS model's scattered intensity per unit volume, particularly for the mesostructure component in OMS, can be described by the following equation:

$$I_{\text{meso}}(q) = (\Delta\rho)^2 n_d \langle F(q)^2 \rangle S(q); \tag{S1}$$

$$S(q) = 1 + \beta(q)(< Z(q) > -1)G(q) \tag{S2}$$

where:

- $\Delta\rho$ represents the scattering contrast between the surrounding environment and the mesoporous material.
- n_d is the number density of the mesoporous particles within the sample.
- $\langle F(q)^2 \rangle$ denotes the average form factor squared, capturing the shape and size effects of the particles.
- $S(q)$ is the structure factor, which accounts for the interference effects due to the spatial arrangement of the particles.

Furthermore, $n_d(\Delta\rho)^2$ effectively acts as the scale factor Sc_{meso}, linking the mesoscopic scattering intensity to the atomic structural details captured by the form and structure factors.
The square of form factor $P(q) =< F(q) >^2$ for long cylinder can be approximately factorized into longitudinal and cross-section contribution under the condition that the length of the cylinder is much larger than the radius. Thus $P(q)$ becomes

$$P(q) = P_{rod}(q)P_{CS}(q) \tag{S3}$$

Then, the longitudinal factor $P_{rod}(q)$ is taken as the form factor of an infinitely long thin rod:

$$P_{rod}(q) = (\frac{2Si(qL)}{qL} - \frac{4\sin^2\frac{qL}{2}}{(qL)^2})^2 \tag{S4}$$

where

$$Si(x) = \int_0^x t^{-1} \sin t \, dt \tag{S5}$$

and L and R are the length and radius of the cylinder[1]. In the implementation of the model fitting, the L will be fixed in 5000. The cross-section contribution $P_{CS}(q)$ is

$$P_{CS}(q) = (\frac{2B_1(qR)}{qR})^2 \tag{S6}$$

where $B_1(x)$ is the first order of the Bessel function. In the model, we consider the mesopres are core-shell cylinder shape, and scattering length density profile change gradually from the empty of the skeletal material. Thus, the cross-section form factor $F_{CS}(q)$ is described by[2]

$$F_{CS}(q) = r_{\text{out}}^2 \frac{2B_1(qr_{\text{out}})}{qr_{\text{out}}} S_{\text{smear}}(q) - \left(1 - \frac{\Delta\rho_{\text{in}}}{\Delta\rho_{\text{out}}}\right) r_{\text{in}}^2 \frac{2B_1(qr_{\text{in}})}{qr_{\text{in}}} \tag{S7}$$

$$r_{\text{out}} = r_{\text{in}} + t \tag{S8}$$

$$S_{\text{smear}}(q) = \exp\left(-\frac{1}{2}q^2\sigma_{\text{out}}^2\right) \tag{S9}$$

where $\Delta\rho_{\text{in}}/\Delta\rho_{\text{out}}$ is the ratio between the inner and outer electron density contrasts of the cylinder, r_{out}, r_{in}, and t are the outer, inner radii, and thickness of the cylinder shell, respectively. The Gaussian smearing functions $S_{\text{smear}}(q)$ is used to account for roughness at the outer surfaces. Larger values of σ indicate a more gradual change in the scattering length density. Z(q) is the lattice factor describing the spatial distribution of the particles, and the angular brackets $< >$ means an average operation in terms of the particle size and spatial distribution of particles, which are assumed to be independent. These two equations imply a decoupling approach that allows one to factorize the scattering intensity into the contributions from the form factor and the structure factor.

Using the Miller indices (h k l) for a given crystal lattice plane, the lattice factor for an ideal undistorted lattice, $Z(q)$ has the expression [3]:

$$Z(q) = \frac{(2\pi)^{d-1}c}{nv_d\Omega_d q^{d-1}} \sum_{hkl} m_{hkl} f_{hkl}^2 L_{hkl}(q) \tag{S10}$$

where n is the number of particles per unit cell, v_d is the volume (d=3), surface (d=2), or long-period (d=1) of the d-dimensional unit cell, Ω_d is the d-dimensional solid angle, f_{hkl} is the symmetry factor that takes into account symmetry-related extinction rules, $L_{hkl(q)}$ is a normalized peak shape function, and m_{hkl} is the reflection multiplicity.

The mesopores in the SBA-15 are in a p6mm space group, 2-D hexagonal lattice structure, so Z(q) can be written in this format:

$$Z_0(q) = c\frac{2}{\sqrt{3}a^2q} \sum_{hk} m_{hk} L_{hk}(q) \tag{S11}$$

Here, c is a constant of order unity, which ensures that the product of form factor and structure factor fulfills the equation for Porod invariant Q, a is the lattice parameter, and m_{hk} is the multiplicity factor of the hexagonal lattice with values of 12 ($for\, h \neq k \neq 0$) and 6. Porod invariant Q is the integral over all **q** of the intensity, and it is given by

$$Q = \int_0^\infty q^2 I(q)dq \tag{S12}$$

In the q range of interest, there are five obvious peaks in the SAXS data of the 2d hexagonal corresponding Miller indies are (10), (11), (20), (21), and (30). L_{hk} is the peak shape functions, and they have the expression:

$$L_{hk}(q) = \frac{2}{\pi\delta}\left|\frac{\Gamma[\frac{\nu}{2} + \frac{i\gamma_\nu 2(q-q_{hk})}{\pi\delta}]}{\Gamma[\frac{\nu}{2}]}\right|^2, \int_{-\infty}^{+\infty} L_{hk}(q)\,dq = 1 \tag{S13}$$

where $\Gamma[z]$ is a complex gamma function, q_{hk} determines the peak position, and ν controls the shape of the peaks. In the limit $\nu \to 0$, the peak function L_{hk} is a Lorentzian form, and $\nu \to \infty$, it is a Gaussian form. δ is the width of the peak, which is related to the domain size D of the ordered domain through the Dybye-Scherrer function[2]:

$$D = \frac{2\pi}{\delta} \tag{S14}$$

G(q) is similar to the Debye-Waller factor, which describes the disorder of the lattice, and given by

$$G(q) = e^{-(\sigma_a a q)^2} \tag{S15}$$

$\beta(q)$ includes the effect of the polydispersity of the cylinders in their radii to the interference term, and its expression is

$$\beta(q) = \frac{< F_{CS}(q) >^2}{< F_{CS}(q)^2 >} \tag{S16}$$

$$< F_{CS}(q) > = \frac{\int_0^\infty D(r) F_{CS}(q,r)\, dr}{\int_0^\infty D(r)\, dr} \tag{S17}$$

$$< F_{CS}(q)^2 > = \frac{\int_0^\infty D(r) F_{CS}(q,r)^2\, dr}{\int_0^\infty D(r)\, dr} \tag{S18}$$

In the equation, the brackets $< ... >$ means the average of all possible cylinder sizes, weighted by a distribution D(r), which in our model is Gaussian distribution, which expression is given by

$$D(r) = \frac{1}{Norm} \exp\left(-\frac{(r - r_{med})^2}{2\sigma_r^2}\right) \tag{S19}$$

where r_{med} is the median value of the Gaussian distribution, σ is a parameter describing the width of the underlying normal distribution, and Norm is the normalization factor. In the model $pd = \frac{\sigma_r}{r_{med}}$. In the actual numerical computation, we will use 80 points and $\pm 8\sigma$ range (the minimum value of the r = 0). In the implementation of this model, we only add the polydispersity into the inner radii, because the intensity pattern are very similar even if the polydispersity into the thickness t of the cylinder shell is added. In the high q range, the scattering intensity pattern is dominated by the microstructure, and it can be described by the following function: [4]

$$I_{micro}(q) = Sc_{micro} \frac{\xi^3}{(1 + (q\xi)^2)^2} \tag{S20}$$

where ξ is the correlation length and interpreted as diameter of the micropores, and Sc_{micro} is the scale factor of the micropore which is related to the volume fraction of the micropore. According to Porod's law, $I_{Porod}(q)$ is only contributing in the low q-range ($q < 0.01^{-1}$) and comes from interface between the grains:

$$I_{Porod}(q) \simeq \frac{Sc_{macro}}{q^d} \tag{S21}$$

$$Sc_{macro} = 2\pi(\Delta\rho)^2 S_v \tag{S22}$$

where Sc_{macro} is the scale factor for Porod's law, and S_v the specific surface area (i.e. surface area/volume) of the material. The fitting parameters are summarized in the Table S1. Thus, the final expression of the model is

$$\begin{aligned} I(q) = Sc_{meso} P_{rod}(q) < F_{CS}(q)^2 > (1 + \beta(q)[< Z(q) > -1] G(q)) \\ + I_{micro}(q) + I_{Porod}(q) + bkg \end{aligned} \tag{S23}$$

where bkg term accounts for the background intensity.

Table S1. Summary of all parameters contained in the multi-scale model.

Parameter	Description
Sc_{meso}	scale for the mesoporous structure contribution
Sc_{micro}	scale for the micropore scattering contribution
Sc_{macro}	scale for the macrospore contribution
a	lattice parameter
δ	full width at half maximum related to the domain size
σ_a	lattice disorder parameter
r	the mean radius
t	thickness of the cylinder
$\frac{\Delta\rho_{in}}{\Delta\rho_{out}}$	ratio between inner and outer contrast, for the empty pore, $\frac{\Delta\rho_{in}}{\Delta\rho_{out}} = 0$
σ_{out}	smearing width of the outer shell
pd	relative polydispersity of radii
ξ	the correlation length of micropores
bkg	the background intensity
L	length of the cylinder (will be fixed in 5000 Å during analysis)
d	Porod's exponent
ν	peak shape

S2. Contrast Variation Simulation

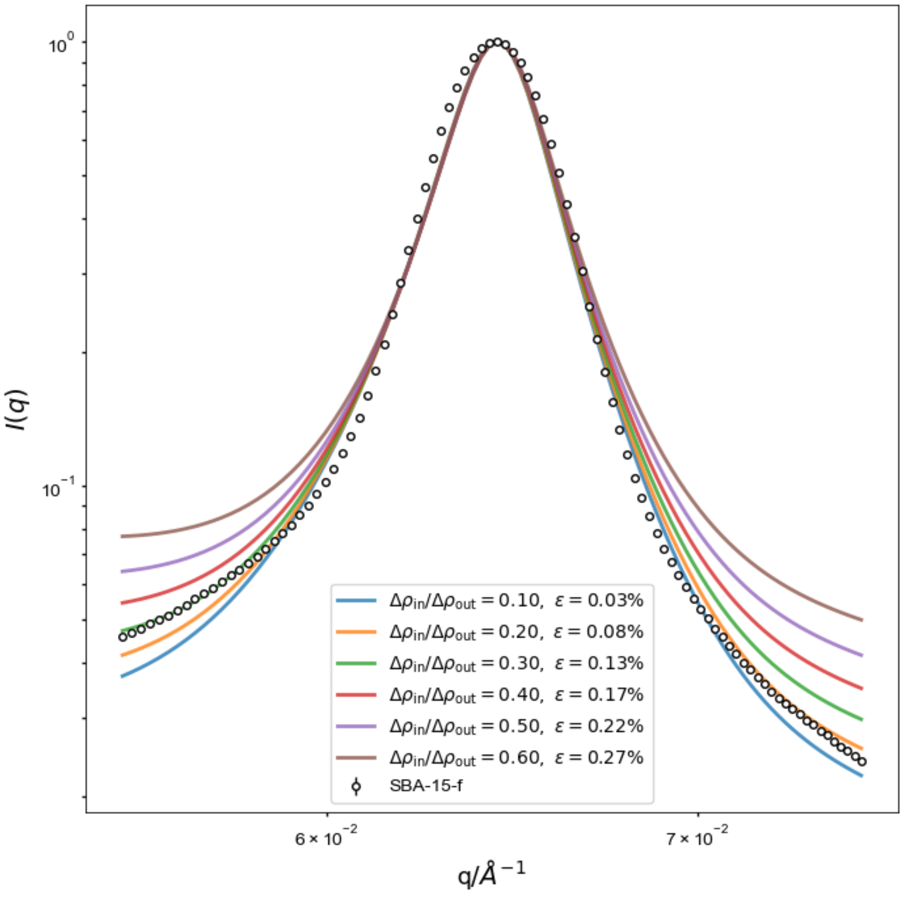

Figure S1. Small-angle X-ray scattering (SAXS) patterns of SBA-15-f of the first peak, and the contrast changes inside the mesopore, and the corresponding strain relative of the experimental data.

S3. SAXS Curves of Degassed Samples

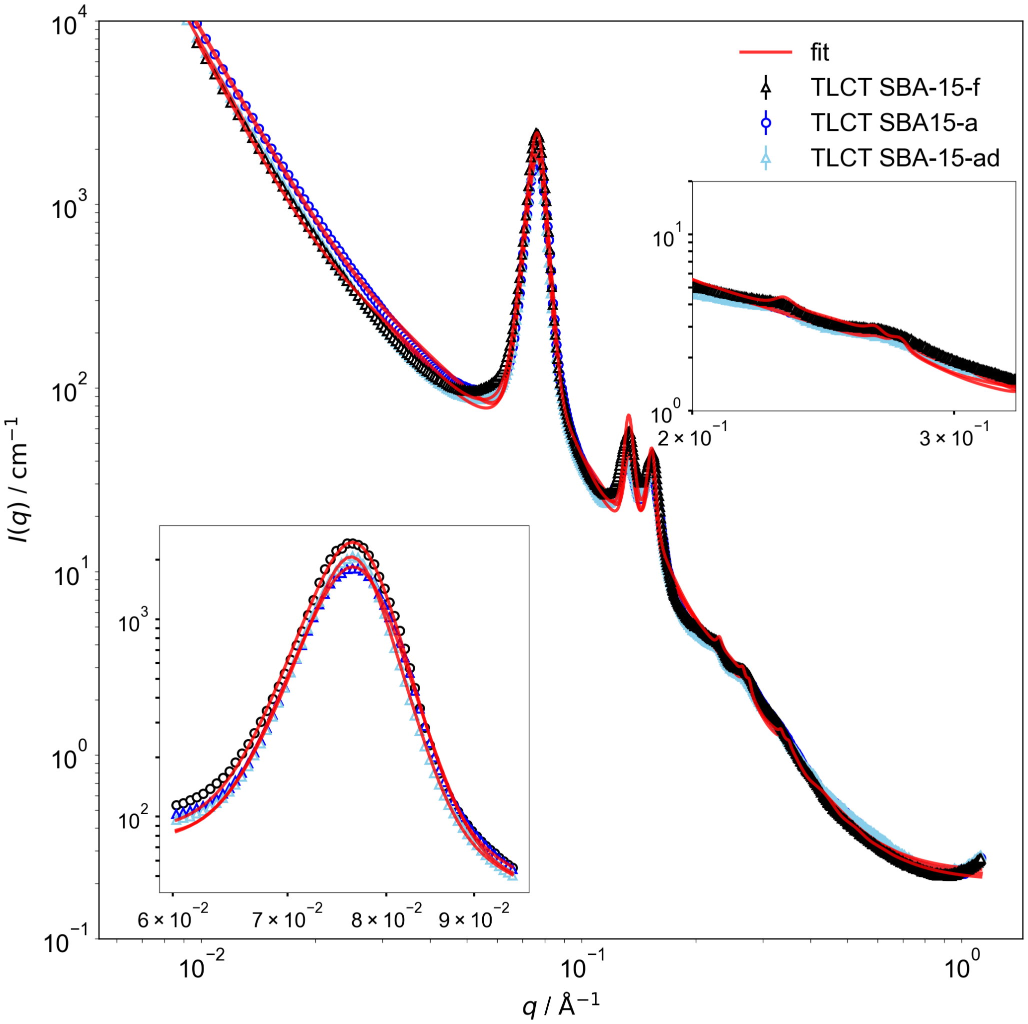

Figure S2. Small-angle X-ray scattering (SAXS) patterns illustrating the aging behaviour of mesoporous SBA-15 synthesized via true liquid-crystal templating (TLCT). The absolute scattered intensity, $I(q)$, is plotted as a function of the magnitude of the scattering vector, q.

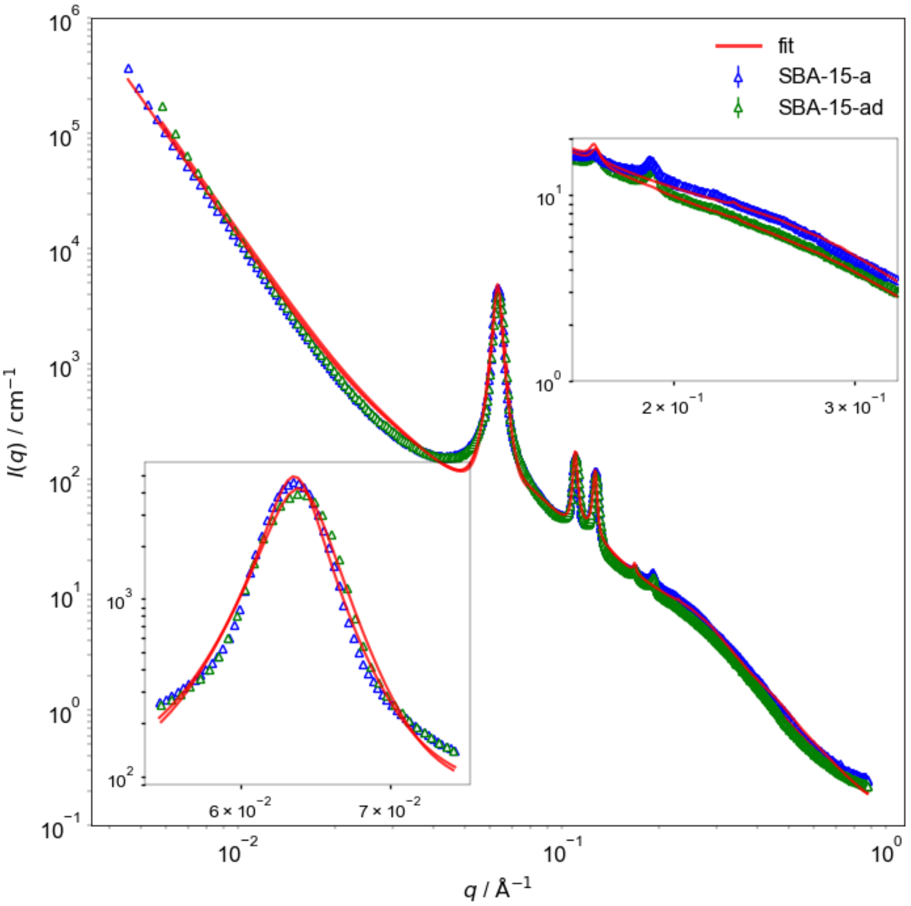

Figure S3. Small-angle X-ray scattering (SAXS) patterns illustrating the aging behaviour of mesoporous SBA-15 synthesized via liquid-crystal templating (LCT). The absolute scattered intensity, $I(q)$, is plotted as a function of the magnitude of the scattering vector, q. Data for SBA-15-a was gently dried under a constant flow of dry N_2 at 50°C for 24 h. SBA-15-ad are shown as green triangles, and those for the aged sample SBA-15-a as blue triangles. All data sets were fitted with the SBA-15 model, and the corresponding model curves are shown as red solid lines. The left inset highlights the shift of the (10) Bragg reflection upon aging. The right inset magnifies the high-q region dominated by micropore scattering.

S4. Sample to Detector Distances

The following Table S2 shows the sample-to-detector (SD) distances for different samples; the different SDs are to cover the wide q range and to achieve improved resolution in q. The exposure time for every SD is one hour.

Table S2. Sample names and corresponding sample-to-detector (SD) distances used in SAXS measurements.

Sample	SD (mm)
LCT SBA-15-f	50, 450, and 1450
LCT SBA-15-a	150, 450, and 900
LCT SBA-15-ad	150 and 550
TLCT SBA-15-f	150 and 550
TLCT SBA-15-a	150 and 550
TLCT SBA-15-ad	150 and 550

S5. Summary of all Structural Parameter Values from the SAXS Model

Table S3. Summary of multi-scale SAXS model parameters for various mesoporous silica samples.

**Sample**	Sc_{meso}	Sc_{micro}	Sc_{macro}	a(Å)	δ	σ_a	r(Å)	t(Å)	σ_{out}	pd	ξ(Å)	bkg	d	ν
LCT SBA-15-f	6.9×10^5	0.16	2.5×10^{-4}	112.6	2.5×10^{-3}	0.05	42.4	29.2	8.7	0.06	13	0.14	4.0	0.35
LCT SBA-15-a	4.2×10^5	0.34	1.3×10^{-4}	114.3	3.1×10^{-3}	0.04	39.1	33.5	2.5	0.12	5.1	0.10	4.0	0.20
LCT SBA-15-ad	4.0×10^5	0.28	1.3×10^{-4}	113.7	3.5×10^{-3}	0.04	40.1	32.3	4.0	0.12	5.6	0.12	4.0	0.35
TLCT SBA-15-f	5.5×10^5	0.12	9.9×10^{-5}	94.5	5.8×10^{-3}	0.05	33.9	26.4	10.0	0.06	9.3	0.21	3.9	1.3
TLCT SBA-15-a	3.7×10^5	0.12	5.7×10^{-4}	94.6	6.3×10^{-3}	0.04	31.0	28.8	6.4	0.09	8.2	0.22	3.6	1.3
TLCT SBA-15-ad	3.6×10^5	0.11	1.6×10^{-4}	94.8	5.5×10^{-3}	0.05	33.4	25.5	10.0	0.07	9.0	0.23	3.8	1.0

S6. Swelling Effect

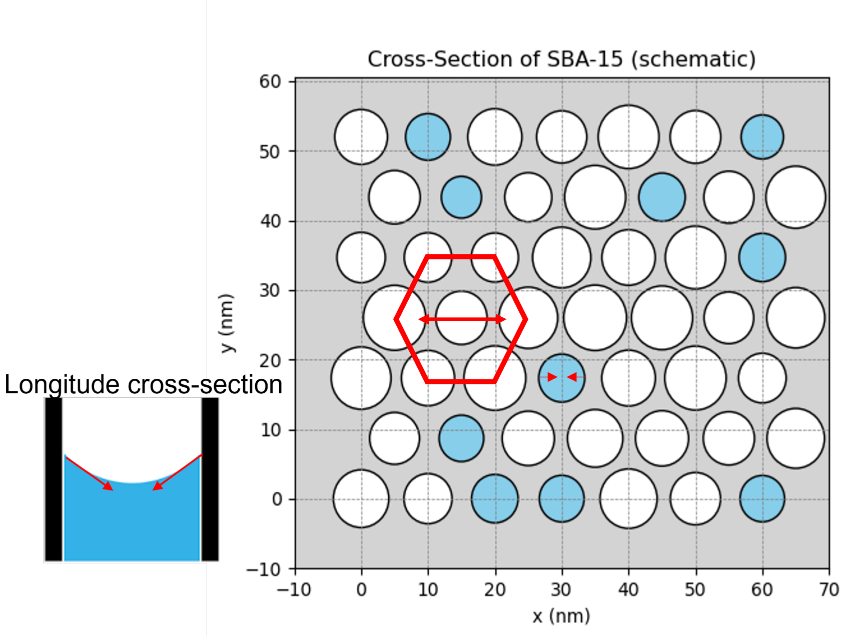

Figure S4. Schematic description of the lattice swelling. The figure on the left shows the longitude cross section of the capillary condensation in cylindrical pore, and the red arrows show the force of the water added on the silica wall. The figure on the right shows the radial cross-section of the SBA-15. The mesopores are polydisperse, and capillary condensation occurs in the small pores firstly leads to the contraction of the pore. In the contrary, it will expand the neighbor lattice (red).

S7. Fitting Decouple

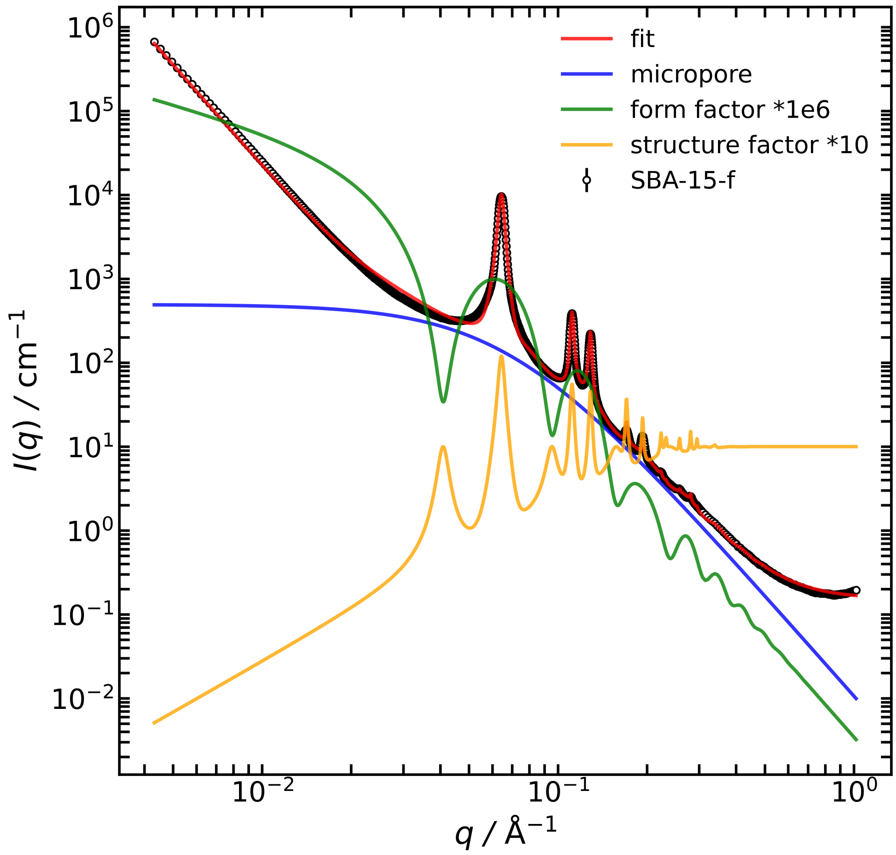

Figure S5. The figure shows the SAXS model fitting (red line) the LCT SBA-15-f (black empty circles) with different contributions, core-shell cylinder form factor plotted in green line, micropores scattering intensity plotted in blue line, and the structure factor plotted in orange line.

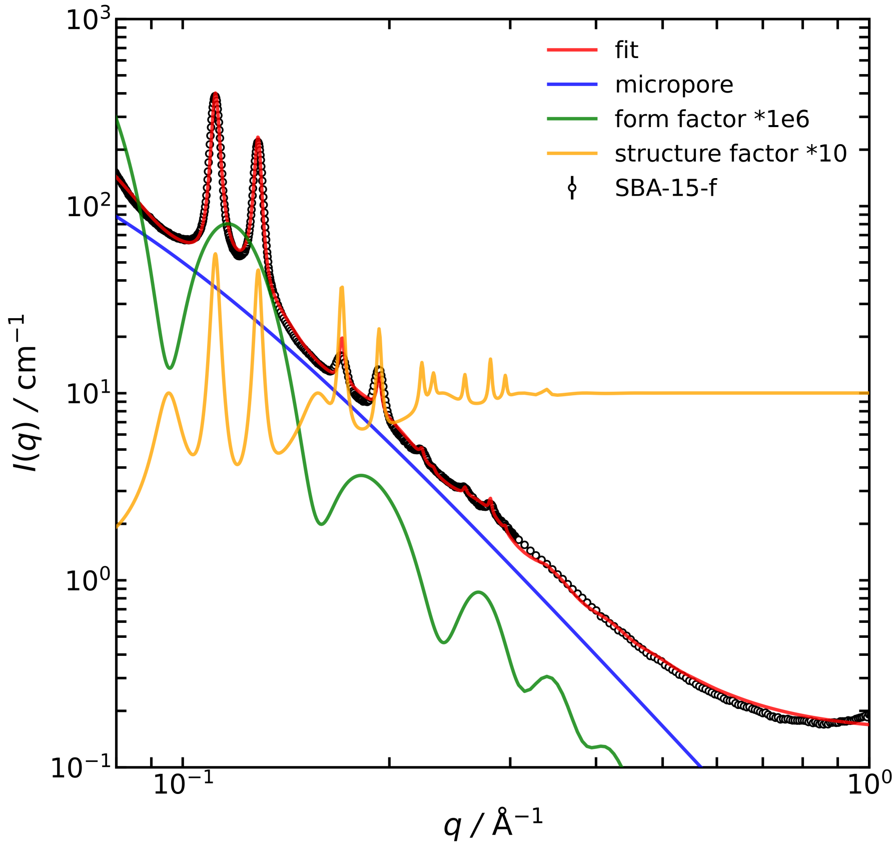

Figure S6. The zoomed of the Figure S5 where $q > 0.08$ Å shows the mismatch of the model and the experimental in (21) and (30) peaks.

S8. Indexing LCT SBA-15 aged with 2D Hexagonal Ordering

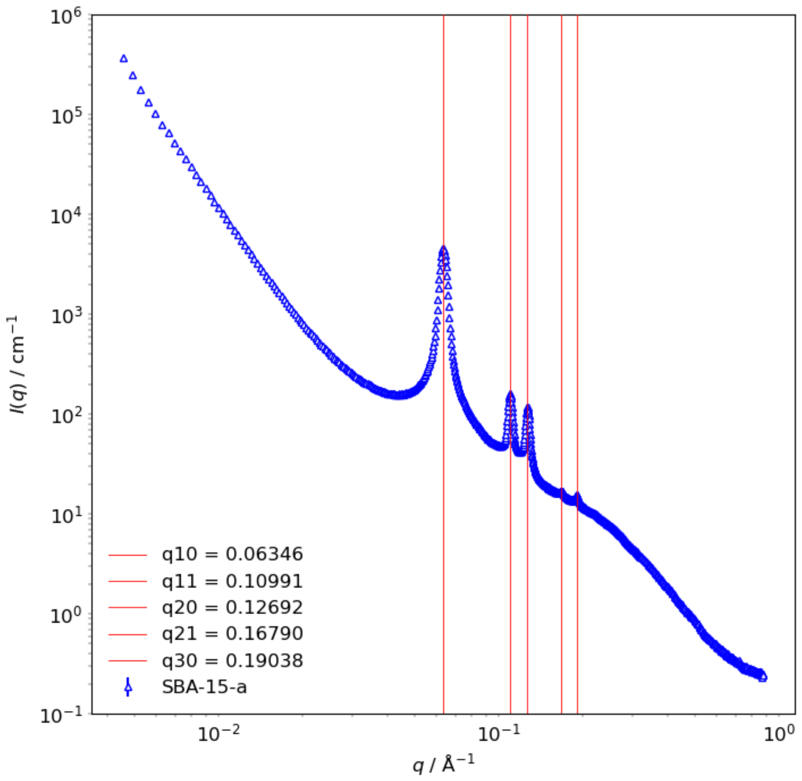

Figure S7. The figure shows the peaks' position and they follow the 2D hexagonal ratio well, which is $1 : \sqrt{3} : 2 : \sqrt{7} : 3$.

S9. Kelvin Radii Table

Table S4. Kelvin radius for capillary condensation calculated from the modified Kelvin equation for cylindrical pores. Values are given in angstrom.

T (°C)	$p/p_0 = 0.40$	$p/p_0 = 0.50$	$p/p_0 = 0.60$	$p/p_0 = 0.70$	$p/p_0 = 0.80$
18	11.85	15.66	21.25	30.43	48.65
19	11.78	15.57	21.13	30.26	48.37
20	11.71	15.48	21.01	30.09	48.10
21	11.65	15.40	20.89	29.92	47.83
22	11.58	15.31	20.78	29.76	47.57
23	11.53	15.24	20.68	29.61	47.33

S10. Error propagation for SAXS peak positions

S10.1. Scattering vector q uncertainty

In small-angle X-ray scattering (SAXS), typically the scattering angle 2θ is in between $0.1 - 10\,^\circ$, and the scattering vector is defined as

$$q = \frac{4\pi \sin\theta}{\lambda}, \tag{S24}$$

where λ is the X-ray wavelength. For small scattering angles ($\sin\theta \approx \frac{\sin 2\theta}{2} \approx \frac{r}{2L}$), the detector geometry gives the approximation

$$q \approx \frac{2\pi r}{\lambda L}, \tag{S25}$$

where r is the radial detector position of the scattering feature and L is the sample-to-detector distance. Therefore, an uncertainty in the effective sample position, for example from finite capillary thickness, gives an approximate relative uncertainty in q:

$$\frac{\sigma_q}{q} \approx \frac{\delta L}{L}. \tag{S26}$$

For the present analysis, the capillary thickness is taken as $\delta L = 0.9$ mm. The geometry contribution to the q-uncertainty is therefore

$$\sigma_{q,\text{geom}} = q\frac{0.9}{L}, \tag{S27}$$

where L is given in mm.

Table S5. Sample-to-detector distances used for the propagation of the geometric q-uncertainty.

Sample group	L (mm)
LCT SBA-15 fresh	1450
LCT SBA-15 aged	900
All other samples	550

S10.2. Uncertainty of the intensity-weighted peak position

The calculated peak position, q_{cal}, is the intensity-weighted peak position inside the selected peak window:

$$q_{\text{cal}} = \frac{\sum_i q_i I_i}{\sum_i I_i}. \tag{S28}$$

This centroid-like peak position depends on all measured intensities in the window. Therefore, intensity uncertainties also contribute to the uncertainty of q_{cal}. Let

$$Q = \frac{\sum_i q_i I_i}{S}, \qquad S = \sum_i I_i, \tag{S29}$$

where $Q = q_{\text{cal}}$. The derivative of Q with respect to an intensity value I_i is

$$\frac{\partial Q}{\partial I_i} = \frac{q_i - Q}{S}. \tag{S30}$$

Assuming independent intensity uncertainties, the intensity-derived contribution is

$$\sigma_{Q,\text{int}} = \sqrt{\sum_i \left[\frac{q_i - Q}{S}\sigma_{I_i}\right]^2}. \tag{S31}$$

The geometry contribution for the intensity-weighted peak position is

$$\sigma_{Q,\text{geom}} = Q\frac{0.9\ mm}{L}. \tag{S32}$$

The total uncertainty is then obtained by adding the independent contributions in quadrature:

$$\sigma_{Q,\text{total}} = \sqrt{\sigma_{Q,\text{int}}^2 + \sigma_{Q,\text{geom}}^2}. \tag{S33}$$

In the exported peak table, the column `q_calculated_uncertainty` reports $\sigma_{Q,\text{total}}$.

S10.3. Strain uncertainty

For two corresponding peaks, the strain is calculated from the intensity-weighted peak positions:

$$\varepsilon = \frac{q_{\text{reference}}}{q_{\text{compared}}} - 1. \tag{S34}$$

The propagated uncertainty is

$$\sigma_{\varepsilon} = \sqrt{\left(\frac{\sigma_{q,\text{reference}}}{q_{\text{compared}}}\right)^2 + \left(\frac{q_{\text{reference}}\sigma_{q,\text{compared}}}{q_{\text{compared}}^2}\right)^2}. \tag{S35}$$

For the Table 3, `average_strain_uncertainty_percent` is calculated from the propagated per-peak strain uncertainties:

$$\sigma_{\bar{\varepsilon}} = \frac{\sqrt{\sum_i \sigma_{\varepsilon_i}^2}}{n}, \tag{S36}$$

where n is the number of peaks used in the average.

1. Pedersen, J.S. Analysis of small-angle scattering data from colloids and polymer solutions: modeling and least-squares fitting. *Advances in Colloid and Interface Science* **1997**, *70*, 171–210.
2. Sundblom, A.; Oliveira, C.L.P.; Palmqvist, A.E.C.; Pedersen, J.S. Modeling in Situ Small-Angle X-ray Scattering Measurements Following the Formation of Mesostructured Silica. *J. Phys. Chem. C* **2009**, *113*, 7706–7713. https://doi.org/10.1021/jp809798c.
3. Förster, S.; Timmann, A.; Konrad, M.; Schellbach, C.; Meyer, A.; Funari, S.S.; Mulvaney, P.; Knott, R. Scattering curves of ordered mesoscopic materials. *J. Phys. Chem. B* **2005**, *109*, 1347–1360. https://doi.org/10.1021/jp0467494.
4. Debye, P.; Anderson, H.R.; Brumberger, H. Scattering by an Inhomogeneous Solid. II. The Correlation Function and Its Application. *J. Appl. Phys.* **1957**, *28*, 679–683. https://doi.org/10.1063/1.1722830.
